# Attenuating pulmonary injury and inflammation with C5/CD14 inhibition therapy: results from a porcine polytrauma model with blunt chest trauma

**DOI:** 10.1007/s00068-026-03157-w

**Published:** 2026-03-24

**Authors:** Ümit Mert, Rald V. M. Groven, Kang Qin, Johannes Greven, Elizabeth Rosado Balmayor, Tom Eirik Mollnes, Markus Huber-Lang, Martijn van Griensven, Frank Hildebrand, Klemens Horst

**Affiliations:** 1 Department of Orthopedic, Trauma- and Reconstructive Surgery, RWTH Aachen University Hospital, Pauwelsstraße 30, 52074 Aachen, Germany; 2 Department of Orthopedics and Trauma Surgery, Helios University Hospital, University Witten/Herdecke, Heusnerstraße 40, 42283 Wuppertal, Germany; 3 Department of Cell Biology-Inspired Tissue Engineering, MERLN Institute for Technology- Inspired Regenerative Medicine, Maastricht University, Universiteitssingel 40, Maastricht, 6229 ER The Netherlands; 4 Experimental Orthopedics and Trauma Surgery, Department of Orthopedic, Trauma- and Reconstructive Surgery, RWTH Aachen University Hospital, Pauwelsstraße 30, 52074 Aachen, Germany; 5 Research Laboratory, Nordland Hospital Bodø, Parkveien 95, Bodø, 8005 Norway; 6 Department of Immunology, Oslo University Hospital, University of Oslo, Sognsvannsveien 20, Oslo, 0372 Norway; 7 Institute of Clinical- and Experimental Trauma-Immunology, Ulm University Hospital, Helmholzstraße 8/1, 89081 Ulm, Germany; 8 Clinic for Trauma, Hand, and Reconstructive Surgery, Münster University Hospital, Albert- Schweitzer-Campus 1, 48149 Münster, Germany

**Keywords:** Polytrauma, Lung injury, Immunomodulation, Complement system inhibition, Toll-like receptor signaling inhibition, Inflammation, Alveolar macrophages

## Abstract

**Background:**

Thoracic injury is prevalent among polytrauma patients, affecting up to 45% of these patients and often leading to complications such as pulmonary dysfunction up to acute respiratory distress syndrome (ARDS). This study investigates the impact of surgical invasiveness and C5/CD14 inhibition therapy on pulmonary tissue damage and the local immune response of the lungs in a porcine polytrauma model.

**Methods:**

A *post hoc* analysis focusing on the pulmonary consequences of blunt thoracic trauma and multiple trauma was performed in a previously published porcine trauma model, in presence or absence of immunomodulation. A control group (*n* = 6; CG) was used that received identical instrumentation, anesthesia, nutrition, and fluid intake, but no trauma. For the trauma model, male pigs underwent a standardized polytrauma, followed by allocation into three treatment groups: early total care (*n* = 8; ETC), damage control orthopedics (*n* = 8; DCO), and ETC with C5/CD14 inhibition (*n* = 4). Pulmonary histopathological changes were assessed at 72 h after trauma through lung injury scores and wet/dry ratios, while ex vivo cytokine expressions from isolated alveolar macrophages (AMs) were analyzed using enzyme-linked immunosorbent assays.

**Results:**

Histological results revealed significant lung damage in both the ETC and DCO groups compared to the control as well as the C5/CD14 inhibition group. The addition of C5/CD14 inhibition significantly lessened lung tissue damage, as indicated by lower lung injury scores and wet/dry ratios. Cytokine assays showed that AMs from the ETC + C5/CD14 group exhibited significantly decreased levels of proinflammatory cytokines compared to the other trauma groups, while showing similar cytokine expression levels as compared to the control group.

**Conclusions:**

Early double blockade of C5/CD14 effectively mitigates pulmonary tissue damage and post-traumatic inflammatory responses in lung tissue. This might result in reduced complications like ARDS and facilitating earlier definitive surgical interventions. These findings underscore the potential importance of immunomodulation strategies in managing post-traumatic outcomes and highlight the need for further research into their clinical applications in polytrauma settings.

## Introduction

Up to 45% of all polytrauma patients sustain thoracic injury, making it one of the most prevalent injuries in the polytraumatized patient [1]. Apart from the direct impact, local immune responses in the lung contribute to the development of severe complications such as acute respiratory distress syndrome (ARDS) over time. Despite efforts to prevent these complications and improve their treatment outcome [2], morbidity and mortality rates in trauma patients remain high [3, 4]. Apart from the trauma itself, the type of surgical treatment and its timing are also essential in enabling and potentially maintaining immunological homeostasis, as well as preventing the occurrence of complications related to an imbalanced post-traumatic immune response, such as remote organ damage [5]. 

In past decades, research in the field of trauma has therefore focused on different treatment concepts, such as early total care (ETC) and damage control orthopedics (DCO), which aim at guiding surgical interventions against the background of the patients immunological status [6]. The main aim of these concepts is to perform surgery as soon as safely possible, considering the magnitude of the surgery and the patient’s immunological status, in an effort to enable early patient mobilization and recovery. Regulating and balancing the immune response, in particular in the early post-traumatic phase, is therefore of significant clinical interest to reduce the occurrence of secondary pulmonary inflammatory complications and enhance patient outcome by enabling earlier primary definitive surgical care (7).

With regard to these post-traumatic inflammatory processes in the lungs, alveolar macrophages (AMs) are among the immune cells of interest. AMs contribute to tissue homeostasis and host defense. They can clear cell debris and repair damaged tissue, recognize various pathogens, and initiate and resolve lung inflammation [8]. Of the two proposed AM phenotypes (M1 and M2), M1 macrophages represent the proinflammatory type and are typically triggered in response to proinflammatory cytokines. This enhances further inflammatory cytokine release and recruitment of immune cells into the lung parenchyma and alveoli. In contrast, M2 macrophages are more regenerative, as characterized by the release of antiinflammatory mediators, such as interleukin 10 [9].

Other two key players that contribute to the development of lung tissue damage and subsequent pulmonary dysfunctions in the acute phase after trauma are Toll-like receptor (TLR) signalling pathway and the complement cascade. TLR4-dependent inflammatory responses have shown to be essential in the development of ARDS in murine models [10]. The CD14 antigen serves as a decisive cofactor for numerous TLRs and plays an important role in the identification of pathogen-associated and damage-associated molecular patterns (PAMPs and DAMPs) [11]. Blocking CD14 prevents the dimerisation of the receptor, halting the pro-inflammatory signal transduction into the cell. Also the complement activation product C5a can trigger various inflammatory activities, including cellular activation and leukocyte chemotaxis (12). Previous studies investigated the role of C5a in injury, confirming the protein’s key role in the development of post-traumatic inflammation [13]. In injured individuals, concentrations of complement activation products have shown to be increased and are linked to the development of complications as well as mortality [14, 15].

Furthermore, synergistic effects between several TLRs and C5a have been reported, leading to more pronounced TLR-mediated inflammatory reactions [16]. A combined inhibition in the form of a CD14 inhibitor and a blockade of the central complement component C5, might therefore be beneficial in preventing dysregulated post-traumatic immune reactions. Despite the fact that such a combined inhibition has shown promising results in sepsis and trauma research [17–22], research on its application and potential benefits in regard to pulmonary complications in the polytrauma setting is scarce, hindering its clinical translation.

Therefore, we defined two aims using a porcine polytrauma model: (1) to determine the effect of surgical invasiveness (ETC vs. DCO) and the addition of C5/CD14 inhibition therapy to ETC treatment on the lung parenchyma, (2) to investigate the effects of polytrauma, different surgical treatments, and C5/CD14 inhibition therapy on the expression of key immunological cytokines from alveolar macrophages harvested from a porcine polytrauma model.

## Materials and methods

### Animals and experimental design

This manuscript reports on original research data gathered in the context of a larger previously published study in accordance with the 3R principles for replacing, refining, and reducing the use of animals in research [20]. The hypotheses and ex vivo and in vitro experiments from the present study were a priori determined. The German governmental office of animal care and use (LANUV) approved the protocols and procedures of the present study (permit number: AZ 81.02.04.2020.A215), which was carried out in accordance with the German Animal Welfare Act. All sections of the present manuscript adhere to the ARRIVE guidelines for reporting on animal research [23]. Male pigs (*n* = 26, *sus scrofa*) of the German landrace aged between 12 and 16 weeks and weighing 35 ± 5 kg were used for this study. Immediately after arrival to our Institute for Laboratory Animal science, animals were examined by a veterinarian after which they were housed for seven days before to the start of the experiments to acclimatize. Sample sizes for the sham, ETC, and DCO groups were calculated a priori via a power calculation with 80% power. Animals were randomly allocated to one of four experimental groups: control group (CG; *n* = 6), early total care (ETC; *n* = 8), damage control orthopedics (DCO; *n* = 8), and ETC receiving C5/CD14 inhibition (ETC + C5/CD14; *n* = 4). The latter group was conducted as a pilot group due to the limited availability C5 inhibitor as well as the fact that ETC is expected to elicit increased systemic inflammation and pulmonary tissue damage compared to DCO, making the application of C5/CD14 inhibition most clinically relevant in the ETC group. The control group received identical instrumentation, anesthesia, mechanical ventilation, and parental nutrition (Aminoven; Fresenius SE & Co. KGaA, Homburg, Germany) like in the other experimental groups but was not subjected to trauma.

### Preparation and anesthesia

The animal model was previously described in detail elsewhere (24; 25). In brief: animals were premedicated via intramuscular injection of azaperone (3 to 4 mg/kgBW) (Stresnil™, Janssen-Cilag GmbH, Neuss, Germany) and ketamine (15 mg/kgBW) (Ketanest, Pfizer, New York, NY). This was followed by anesthesia induction with propofol (1 to 2 mg/kgBW) (Fresenius SE & Co. KGaA, Homburg, Germany) after which animals were orotrachealy intubated and ventilated (volume controlled) with a tidal volume of 8–12 ml/kg body weight (BW), a positive end expiratory pressure of 8 mmHg and a plateau pressure of < 28 mmHg (Draeger Evita 4, Draeger Safety AG & Co. KGaA, Lübeck, Germany). Immediately afterwards, electrocardiogram, pulseoximetry, and temperature monitoring was established. General anesthesia was maintained throughout the whole experiment with propofol (5 to 10 mg/kgBW/hr) (Fresenius SE & Co. KGaA, Homburg, Germany) and Midazolam (0.03 to 0.2 mg/kgBW/hr) (Panpharma GmbH, Trittau, Germany). Fentanyl (1 µg/kgBW/hr) (Panpharma GmbH, Trittau, Germany) was used for analgesia. Continuous crystalloid infusions (2 ml/kgBW/hr) (Sterofundin ISO^®^, B. Braun AG, Melsungen, Germany) ensured adequate fluid balance [24]. A central venous catheter (Four-Lumen Catheter, 8.5 Fr., Arrow Catheter, Teleflex Medical GmbH, Fellbach, Germany) was placed in the external jugular vein for the administration of drugs, blood sampling, and continuous monitoring of the central venous pressure. In order to induce controlled hemorrhage, the right femoral vein was instrumented with a three-lumen hemodialysis catheter (12.0 Fr., Arrow Catheter, Teleflex Medical GmbH, Fellbach, Germany). An arterial line (Vygon GmbH & Co. KG, Aachen, Germany) was placed in the femoral artery for the constant monitoring of invasive blood pressure as well as blood sampling. Finally, a suprapubic catheter (12.0 Fr, Cystofix^®^, B. Braun AG, Melsungen, Germany) was placed for collecting urine [24].

### Polytrauma and surgical procedure

After achieving stable baseline conditions after instrumentation for at least 120 min, animals were subjected to the standardized combined polytrauma entailing blunt chest trauma, bilateral femur fractures, a crosswise incision in the liver, and hemorrhagic shock (ISS = 27) [25]. Before polytrauma induction, oxygen (FiO_2_) was reduced to 0.21 to simulate ambient air. Furthermore, the animals were not prevented from cooling down and fluid administration was stopped. Blunt chest trauma was applied to the inflated lung by shooting a bolt gun machine (Blitz-Kerner, turbocut Jopp GmbH, Bad Neustadt an der Saale, Germany) on a set of two metal plates (1 cm lead and 0.8 cm steel) placed on the dorsocaudal side of the right hemithorax. Thereafter, the same bolt gun machine was used to induce open bilateral femoral shaft fractures by using a T-shaped punch which was placed on the middle of the femur. A midline laparotomy was performed to induce a 4.5 × 4.5 cm crosswise incision in the left liver lobe halfway through the tissues depth to add the aspect of uncontrolled bleeding. The incision was left to bleed uncontrolled for 30 s and then packed with sterile gauze afterwhich the abdomen was closed. Subsequently, hemorrhagic shock was induced by controlled withdrawal of a maximum of 45% the total blood volume with a target mean arterial pressure of 40 ± 5 mmHg. The polytrauma was then left untreated for a duration of 1.5 h after which the animals were resuscitated according to current trauma guidelines (ATLS^®^, AWMF-S3 guidelines for the treatment of patients with severe and multiple injuries^®^): re-transfusion of the withdrawn and warmed blood (Citrate Phosphate Dextrose Adenine DONOpacks, Lmb Technologie GmbH, Oberding, Germany) took place as well as crystalloid fluid infusion (Sterofundin ISO^®^, B. Braun AG, Melsungen, Germany). Normothermia (38.7–39.8 °C) was reinstated and kept using a forced air warming system and blankets.

After resuscitation, operative stabilization of the bilateral femur fractures was performed by intramedullary nailing for the ETC and ETC + C5/CD14 inhibition groups (T2-System; Stryker GmbH & Co. KG, Duisburg, Germany) or external fixation for the DCO group (Radiolucent Fixator, Orthofix, Texas, USA) under sterile conditions using fluoroscopy (Ziehm Vision, Ziehm Imaging, Nuremberg, Germany). All procedures were performed by the same experienced surgeon (ÜM). Two grams of the antibiotic ceftriaxone (ceftriaxone^®^ 2 g, i.v., Fresenius, Homburg, Germany) were given before the start of the operation and every 24 h thereafter. Animals were treated according to current ICU guidelines for 72 h before sacrifice and turned every 4-6 h to support respiratory mechanics.

### C5/CD14 inhibition therapy

Animals were allocated according to the groups listed above. CD14 blockade was achieved by recombinant anti-CD14 antibody rMil2 (clone MIL2; isotype IgG2a), produced as an IgG2/4 chimera in the laboratory of Professor T.E. Mollnes [26] and manufactured large scale by ExcellGene SA (Monthey, Switzerland) according to GMP standards. Aside blockade of CD14, the anti-CD14 has no other known effector functions. The C5 inhibitor RA101295 (2-kDa- peptide; provided by UCB, Brussels, Belgium) blocks the cleavage of C5 into C5a and C5b, and thus also the formation of C5b-9 complement complex. Due to the limited access to C5 inhibitor, the novel immunemodulatory therapy was applied in combination with the ETC treatment only. The first ETC + C5/CD14 animal served as reference and received a bolus of anti-CD14 inhibitor (5 mg/kgBW) and a bolus of C5 inhibitor (3 mg/kgBW) 30 min after polytrauma. Following the C5 inhibitor bolus, a continuous infusion (0.55 mg/kgBW/h) was given for up to 64 h after polytrauma induction. The doses for the remaining three pigs were as follows [20]: anti-CD14 bolus (5 mg/kgBW at 30 min, 12 h and 30 h; 2.5 mg/kgBW after 60 h) and a bolus of the C5 inhibitor (5 mg/kgBW at 30 min after trauma) with subsequent continuous infusion (1.1 mg/kgBW/h) for up to 72 h after trauma. The four pigs in the ETC + C5/CD14 inhibition group were merged into one group based on the negligible variability in the treatment efficacy between the first and the other three pigs (*p* < 0.05 for all parameters both for 3 vs. 8 and 4 vs. 8 pigs) [20].

### Histology, lung injury score, and wet/dry ratio

Immediately after sacrifice, the lungs were explanted and tissue samples from the right lower dorsal lobe in the area of the pulmonary contusion were harvested. Per animal, one sample was used for the determination of the wet/dry ratio while one samples was directly fixated in 4% paraformaldehyde.

The fixated tissue samples were then embedded in paraffin, sectioned 5 to 8 μm thick, and stained with hematoxylin and eosin to assess histo(patho)logical changes in the lungs as well as for the determination of the lung injury score (LIS; Table 1) [27]. A 200x magnification with an optical microscope (Carl Zeiss, Jena, Germany) was used by two independent investigators to determine the LIS, according to the standards recommended by the American Thoracic Society [27].

Lung tissue edema was assessed by calculating the wet/dry ratio by first weighing the excised lung tissues to obtain the wet weight. The tissue was then heated at 80 °C for 48 h to obtain the dry weight.

**Table 1:** Lung injury scoring system score = [(20 X A) + (14 X B) + (7 X C) + (7 X D) + (2 X E)]/(number of fields X 100)

Parameter	Score per field			
	0	1	2	
Neutrophils in the alveolar space	None	1–5	> 5	
Neutrophils in the interstitial space	None	1–5	> 5	
Hyaline membranes	None	1	> 1	
Proteinaceous debris filling the airspaces	None	1	> 1	
Alveolar septal thickening	< 2x	2x – 4x	> 4x	

### Alveolar macrophage isolation, culture, and cytokine concentrations

Directly after tissue sample harvesting, alveolar macrophages were isolated via bronchoalveolar lavage (BAL) by ligating the right pulmonary bronchus and canulating the left main bronchus. Subsequently, BAL was performed by injecting 5 ml phosphate buffered saline (PBS) into the left main bronchus. After approximately 60 s, the liquid was aspirated. The procedure was repeated 10 times to obtain 50 ml of BAL for the isolation of alveolar macrophages (AMs) for further in vitro experiments. The obtained BAL was centrifuged at 700 x g for 7 min at 4 °C. The cell pellet was resuspended in complete medium (RPMI-1640, 10% fetal bovine serum, 1% penicillin-streptomycin; Thermo Fisher Scientific, Waltham, MA). The AMs were then seeded in six-well plates at a seeding density of 5 × 10^5^ cells/cm^2^ with either complete medium as described above or complete medium with 100 ng/ml lipopolysaccharide (LPS) to trigger and simulate inflammatory activation to see whether the in vivo priming with C5/CD14 inhibition influenced the ex vivo immune response, and subsequently cultured for 24 h (37 °C, 95% humidity and 5% CO_2_). Afterwards, medium was collected and stored at -80 °C for further use. The concentrations of interferon gamma (IFN-γ), tumor necrosis factor alpha (TNF-α), interleukin (IL) 1β, IL-4, IL-6, IL-8, IL-10, and IFN-α in the medium were then determined using enzyme-linked immunosorbent assays according to the manufacturers instructions (Quantikine porcine ELISA kit; R&D systems, Minneapolis, Minnesota, USA).

### Statistical analyses

GraphPad Prism version 9.2.0 (GraphPhad Software, San Diego, CA) was used for statistical analyses and data visualisation. Normality of data distribution was determined using the Shapiro-Wilk test. Subsequently, either a two-way ANOVA followed by Tukey’s *post hoc* test, or a Kruskal-Wallis test was employed, as appropriate. The presentation of data includes the mean or median, supplemented by the standard error of the mean (SEM) or interquartile range (IQR) as applicable. Pairwise comparisons were performed using Mann-Whitney-U tests. For all analyses, the significance limit was set at *p* ≤ 0.05.

## Results

All animals in the study survived until the end of the experiment, except for one animal in the ETC group which succumbed to cardiorespiratory failure after 60 h, and was therefore excluded from further analyses [20].

### Effect of surgical invasiveness and C5/CD14 inhibition on lung tissue damage

**Fig. 1:**
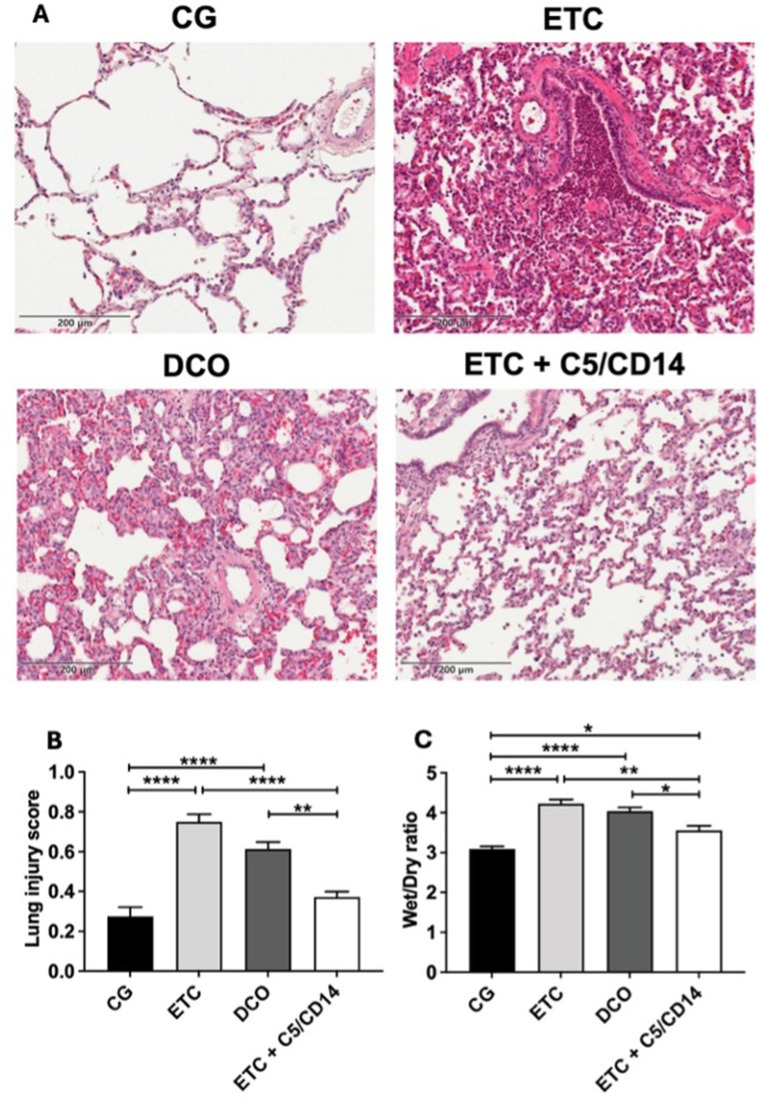
**(A)** Hematoxylin and eosin staining of lung tissue (200 x magnification) from the control (CG), early total care (ETC), damage control orthopedics (DCO), and ETC + C5/CD14 inhibition groups; **(B)** the lung injury scores and **(C) **wet/dry ratios of the four experimental groups. Lung injury scores and wet/dry ratios are depicted as mean, error bars depict standard error of the mean (**** *p* < 0.0001, ** *p* < 0.01, *p ≤ 0.05)

Marked histopathological changes, such as increased exsudations, polymorphonuclear leukocyte infiltrations, overall loss of lung tissue structure, alveolar internal transparent membrane formation, alveolar wall thickening, interstitial edema, and hemorrhage were observed in the lung parenchyma of the ETC group as compared to the CG (Fig. 1A). These histopathological changes were also observed in the DCO group, albeit to a lesser extent as compared to the ETC group. The addition of C5/CD14 inhibition to the ETC treatment resulted in significant reductions of the above mentioned histopathological changes, still exhibiting a diffuse, but reduced infiltration of PMNL. Furthermore, compared to the ETC and DCO groups, more alveolar structures could be recognized in lung tissue from the ETC + C5/CD14 group, which was accompanied by only mild edema.

Based on the these histopathological findings, the LIS showed the most significant increase in the ETC group, followed by the DCO group, compared with the control group (*p* < 0.0001). No significant difference was observed in the LIS between the CG and ETC + C5/CD14 group (Fig. 1B). Furthermore, the addition of C5/CD14 inhibition to the ETC treatment led to significant reductions in the LIS compared to both the ETC group, and the DCO group (*p* < 0.0001 and *p* = 0.004 respectively).

Lastly, the wet/dry ratios also showed significant increases in both the ETC and DCO groups compared to the CG, indicating development of interstitial edema (Fig. 1C; both *p* < 0.0001). The C5/CD14 inhibition therapy combined with ETC significantly alleviated pulmonary edema compared to the ETC (*p* = 0.001) and the DCO groups (*p* = 0.02) (Fig. 1C). However, in comparison to the CG group, the ETC+C5/CD14 exhibited still some edema formation and increase in the wet/dry ratio (*p* = 0.03).

### Cytokine expression from alveolar macrophages

The concentrations of various cytokines secreted by AMs were analyzed in the culture media of all four experimental groups, cultured with and without LPS. Stimulation of the AMs with LPS resulted in marked increases in the concentration of all of the quantified cytokines. Compared to the CG, the AMs harvested from the ETC and DCO groups showed significant increases in the concentrations of TNF-α, IL-1β, IL-4, IL-6, and IL-10.

**Fig. 2:**
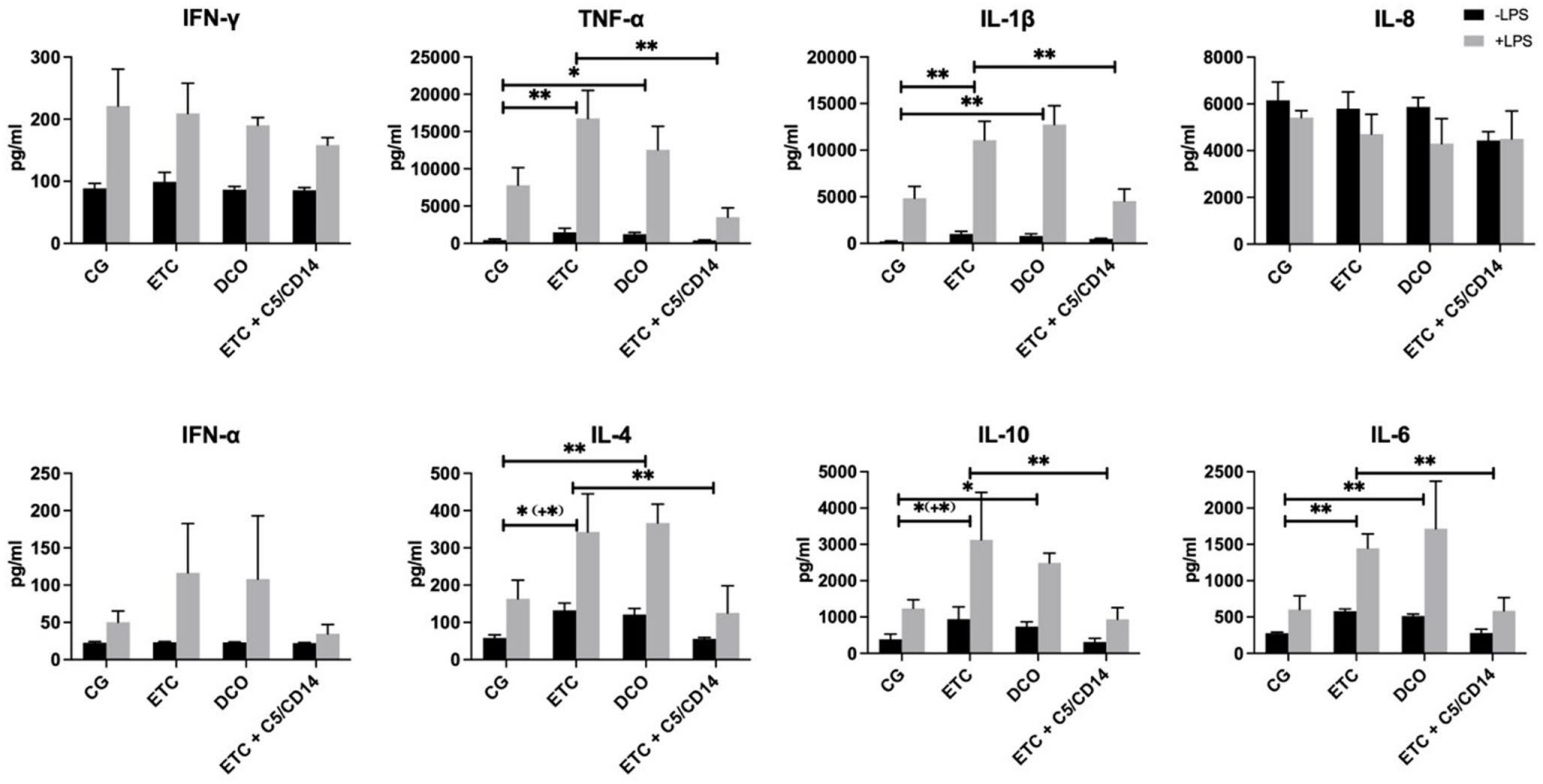
Quantification of cytokine concentrations in the supernatant of alveolar macrophages cultured with or without lipopolysaccharide (LPS; 100ng/ml). Interferon gamma (IFN-γ), tumor necrosis factor alpha (TNF-α), interleukin (IL) 1β, IFN-α, IL-4, IL-6, IL-8, and IL-10concentrations in the control (CG), early total care (ETC), damage control orthopedics (DCO), and ETC+C5/CD14 inhibition groups. The results are expressed in pg/mL, error bars depict standard error of the mean (**p < 0.001, *p ≤ 0.05). For TNF-α, IL-1β and IL-6, significant differences between the experimental groups fell within the same categories of significance (* or **) for alveolar macrophages cultured with or without LPS. For IL-4 and IL-10, the level of significance was higher in alveolar macrophages cultured with LPS than in those cultured without LPS, as indicated by (+*)

Of note, the measured cytokine concentrations from AMs exposed to LPS were significantly less in the ETC + C5/CD14 inhibition group compared to the ETC and DCO groups. Furthermore, no significant differences were observed in the measured cytokine concentrations between the ETC + C5/CD14 inhibition group and the control group. However, no significant differences between the groups were found for IFN-γ, IL-8, and IFN-α (Fig. 2).

## Discussion

Aside from the direct traumatic impact, systemic and local post-traumatic responses can further contribute to the patients’ clinical course and mortality [5]. Modulation of these post-traumatic immune responses may therefore offer great therapeutic potential in reducing the risk of secondary pulmonary complications and eventually may lead to improved patient outcome [28]. Therefore, this study examined the effect of the C5/CD14 inhibition strategy on histopathological changes in the pulmonary parenchyma, and on the secretion of immunological mediators by AMs, using a clinically relevant large animal model of multiple trauma [20]. The main findings are summarized as follows:

- In vivo application of ETC+C5/CD14 inhibition therapy markedly reduced histopathological changes in the lungs, as represented by significant reductions in the lung injury score and wet/dry ratio as compared to the ETC and DCO groups.
- The concentrations of proinflammatory cytokines released from ex vivo cultured AMs were significantly lower in the ETC+C5/CD14 inhibition group as compared to the ETC or DCO groups.
- Moreover, combined C5/CD14 blockade was associated with a reduction in pulmonary damage after ETC treatment as well as a reduction in proinflammatory cytokine release from AMs toward levels observed in the sham group.

Microscopically, distinct histopathological changes in the lung tissue were observed after trauma in both the ETC and DCO groups, such as interstitial edema, septal thickening, and inflammatory cell infiltrations. The ETC treatment’s increased surgical invasiveness elicited significantly more prominent histopathological changes as compared to the DCO group, as illustrated by the derived increased LIS. These findings align with previous studies indicating that the lungs serve as a secondary target in polytrauma [29, 30]. Furthermore, the results from the present study highlight the impact of surgical invasiveness on both systemic and local inflammatory complications [5]. The C5/CD14 inhibition therapy markedly reduced these hispathological changes, as demonstrated by the significant decrease in the LIS in the ETC + C5/CD14 inhibition group as compared to both the ETC and DCO groups. In fact, no significant difference was observed in the LIS between the CG and ETC + C5/CD14 inhibition group. Similar results were observed for the wet/dry ratio, showing that C5/CD14 inhibition significantly lessened pulmonary edema compared with the ETC or DCO groups. The pronounced reduction in inflammatory cell infiltrations in the ETC + C5/CD14 inhibition group is most likely a key factor in the observed preservation of lung tissue [31]. Taken together, these findings are in line with previous research on the application of C5/CD14 inhibition during sepsis, where this immunemodulation resulted in enhanced organ preservation and reduced systemic inflammation [18, 32]. The present study revealed that, also in the polytrauma setting, C5/CD14 inhibition leads to preservation of the lung parenchyma.

In the lungs, AMs play an important role in orchestrating the local post-traumatic immune response, as well as in clearing and regenerating damaged tissue [33]. In relation to pulmonary inflammatory complications, AMs are among the most important cell types that stimulate and induce excessive cytokine release, otherwise known as a cytokine storm [34]. At a biomolecular level, this typically entails the expression of a plethora of inflammatory cytokines from AMs, such as TNF-α, IL-1β, and IL-6 [35, 36]. The findings from the present study are supported by these data, since increased surgical invasiveness markedly enhanced both pro- and antiinflammatory cytokine generation from AMs. Reduced surgical invasiveness, represented by the DCO group, attenuated the increased production of these cytokines but the C5/CD14 inhibition therapy led to the most significant reductions in cytokine concentrations compared to both the ETC and DCO groups. Matching with the histopathological changes described above, no significant differences were observed between the cytokine levels from the CG and ETC + C5/CD14 groups. These results indicate that the early therapeutic blockade of C5/CD14 after trauma may reduce post-traumatic inflammatory complications, and thus theoretically allow for earlier immunological homeostasis and subsequent definitive surgical care, which may even speed up patient recovery [6].

Previous work by Neunaber et al. demonstrated that chest trauma accompanied by a femoral fracture that was surgically treated via intramedullary nailing significantly increased the secretion of TNF-α and IL-6 from AMs, which is in line with our findings [29]. Furthermore, several trauma models, both rodent and porcine, have demonstrated the ameliorating effects of inhibiting complement systems and TLR signalling on acute lung injury and systemic inflammation [17, 37, 38]. For all cytokines except for IL-8, LPS exposure resulted in a significant increase in cytokine release from AMs of the respective treatment group. The cytokine concentrations among the groups was similar as to that of the AMs cultured without LPS and interestingly, the in vivo exposure of the cells to the C5/CD14 inhibition enabled them to effectively dampen the inflammatory response ex vivo. These findings are corroborated by previous work from amongothers Earhart et al. who showed that in vivo priming of AMs in C3-deficient mice led to a reduced cellular chemokine and cytokine output ex vivo [39]. Furthermore, in a non-human primate model by van Griensven et al., it was shown that C3 inhibition improved immune, coagulation, and organ function after traumatic hemorhagic shock [40]. Likewise, in another murine model of pulmonary inflammation, Zahalka et al. demonstrated that in vivo priming of AMs with LPS led to the sensitization and excessive ex vivo cytokine production upon PAMP stimulation [41]. It should be noted that the LPS stimulation in the present study was singular, meaning that future research should focus on the potential effect of endotoxin-tolerance upon repetitive or consistent LPS stimulation in this setting. Interestingly, apart from significant reductions in proinflammatory cytokine generation in the C5/CD14 inhibition group, the levels of IL-4 and IL-10 were also decreased compared to the ETC and DCO groups [32–34]. These results seem paradoxical considering that both IL-4 and IL-10 posses anti-fibrotic and antiinflammatory properties. In fact, several studies investigated the therapeutic potential of IL-10 for the treatment of lung injury, showing promising results [42]. However, as TLR activation is an important driver of IL-10 production, the blockade of this pathway by the CD14 component of the therapy may underlie its reduced expression in the present study [43]. Taken together with the histomorphological results, the reduced production of IL-4 and IL-10 may indicate a feedback mechanism, comprising of a more rapid reestablishment of pulmonary immunological homeostasis followed by a reduced necessity of antiinflammatory cytokines.

The results from the present study are promising, providing valuable insights into histopathological changes as well as underlying biomolecular mechanisms that may contribute to faster patient recovery after polytrauma.

## Conclusions

The present study demonstrated that enhanced surgical invasiveness showed a trend towards increased histopathological changes in the lung parenchyma as well as significantly elevated inflammatory cytokine secretions by alveolar macrophages. The C5/CD14 inhibition therapy effectively reduced histopathological changes in the lung parenchyma, represented by significant decreases in both the lung injury score and wet/dry ratio. Furthermore, significant reductions in the generation of inflammatory cytokines by AMs were observed, with and without LPS stimulation, indicating that C5/CD14 inhibition may succesfully prevent the onset of a proinflammatory cytokine storm after chest trauma. Taken together, these results indicate that the early administration of C5/CD14 inhibition therapy after trauma may reduce post-traumatic pulmonary inflammatory, allowing in principle for earlier primary definitive surgical care to speed up patient recovery.

## Data Availability

The datasets used and/or analysed during the current study are available from the corresponding author on reasonable request.

## Abbreviations

ARDS: Acute Respiratory Distress Syndrome
C5: Complement factor 5
CD14: Cluster of Differentiation 14
ETC: Early Total Care
DCO: Damage Control Orthopedics
AMs: Alveolar Macrophages
TLR: Toll-like receptor
PAMP: Pathogen Associated Molecular Pattern
DAMP: Damage Associated Molecular Pattern
3R: Replacing, Refining, Reducing the use of animals in research
CG: Control Group
LIS: Lung Injury Score
LPS: Lipopolysacharide
IL: Interleukin
IFN: Interferon
TNF: Tumor Necrosis Factor
